# Higher perfusion pressure and pump flow during cardiopulmonary bypass are beneficial for kidney function–a single-centre prospective study

**DOI:** 10.3389/fphys.2024.1257631

**Published:** 2024-02-14

**Authors:** Jakub Udzik, Jerzy Pacholewicz, Andrzej Biskupski, Krzysztof Safranow, Iwona Wojciechowska-Koszko, Paweł Kwiatkowski, Paulina Roszkowska, Karolina Rogulska, Violetta Dziedziejko, Zuzanna Marcinowska, Sebastian Kwiatkowski, Ewa Kwiatkowska

**Affiliations:** ^1^ Cardiac Surgery Department, Pomeranian Medical University, Szczecin, Poland; ^2^ Department of Biochemistry and Medical Chemistry, Pomeranian Medical University, Szczecin, Poland; ^3^ Department of Diagnostic Immunology, Pomeranian Medical University, Szczecin, Poland; ^4^ Department of Obstetrics and Gynecology, Pomeranian Medical University, Szczecin, Poland; ^5^ Department of Nephrology, Transplantology and Internal Medicine, Pomeranian Medical University, Szczecin, Poland

**Keywords:** cardiac surgery, cardiac anesthesia, cardiopulmonary bypass, acute kidney injury, mean arterial pressure

## Abstract

**Background:** Kidneys play an essential role in the circulatory system, regulating blood pressure and intravascular volume. They are also set on maintaining an adequate filtration pressure in the glomerulus. During the CPB, a decrease in systemic blood pressure and hemoglobin concentration may lead to renal ischemia and subsequent acute kidney injury.

**Methods:** One hundred nine adult patients were prospectively enrolled in this study. The intervention in this study was increasing the flow of the CPB pump to reach the target MAP of > 90 mmHg during the procedure. The control group had a standard pump flow of 2.4 L/min/m^2^.

**Results:** Standard pump flow of 2.4 L/min/m^2^ resulted in mean MAP < 90 mmHg during the CPB in most patients in the control group. Maintaining a higher MAP during CPB in this study population did not affect CSA-AKI incidence. However, it increased the intraoperative and postoperative diuresis and decreased renin release associated with CPB. Higher MAP during the CPB did not increase the incidence of cerebrovascular complications after the operation; patients in the highest MAP group had the lowest incidence of postoperative delirium, but the result did not obtain statistical significance.

**Conclusion:** Maintaining MAP > 90 mmHg during the CPB positively impacts intraoperative and postoperative kidney function. It significantly reduces renal hypoperfusion during the procedure compared to MAP < 70 mmHg. MAP > 90 mmHg is safe for the central nervous system, and preliminary results suggest that it may have a beneficial impact on the incidence of postoperative delirium.

## Introduction

Since the introduction of the cardiopulmonary bypass (CPB) technique in 1952, there has been an ongoing debate on the optimal conditions for this procedure. The plethora of functions of the circulatory system is not easily replaced, and many factors need to be considered. In this investigation, the authors focused on the Mean Arterial Pressure (MAP) and its impact on postoperative kidney function. Despite its vital importance to the procedure, this parameter is still widely discussed, and no unequivocal conclusion has been reached regarding its optimal range during CPB (Kunst et al., 2019; Kotani et al., 2022).

Kidneys play an important role in the circulatory system as they regulate the intravascular volume and initiate the Renin-Angiotensin-Aldosterone (RAA) axis response. The kidneys are also set on maintaining an adequate filtration pressure in the glomerulus, even at the cost of subsequent ischemia within the medulla. This dependency is due to the unique anatomy of renal vasculature (Navar et al., 2008). The ultimate branches of renal arteries–the afferent arterioles divide into a number of capillaries, forming the glomerulus’ vascular net. The capillaries then reassemble to create the efferent arterioles. Efferent arterioles have smaller diameters than the afferent arterioles, creating an outwardly directed pressure gradient that is a driving force of the filtration process. After leaving the glomerulus, the efferent arterioles divide again into peritubular capillaries to enable water and ions exchange with the intratubular filtrate. Only a small fraction of the efferent arterioles descends into the renal medulla as vasa recta, providing blood flow to this area.

The renal medulla is a very metabolically active region, as numerous ion pumps in the ascending limb of the Henle loop move the ions in an ATP-dependent manner. However, the blood flow in the medulla is low (Kennedy-et al., 2013) to preserve the osmotic gradient and allow ions and water absorption into the vasa recta. The balance between oxygen demand and supply in the medulla is a fragile one; for the reasons mentioned above, it greatly depends on the blood flow from the glomerulus.

Kidneys have autoregulatory mechanisms that allow for maintaining a stable blood flow and filtration rate in a wide range of MAP. The lower range limit for this autoregulation is 70–80 mmHg, according to most authors (Jefferson et al., 2010; Burke et al., 2014). Below that threshold, the renal blood flow is decreased, and the juxtaglomerular cells of the afferent arteriole begin to release renin (Lappin). Renin is the first enzyme of the RAA axis, and its release aims to raise systemic blood pressure. The RAA system also has a particular effect on renal circulation. Increased renin level leads to a rise in Angiotensin II (Ang II), a potent vasopressor. Ang II constricts both the afferent and the efferent arteriole within the kidney glomerulus, but its effect is always greater on the efferent arteriole. This results in a rise in the effective filtration pressure but also decreases the amount of blood going to the medulla.

There are a few key changes to the circulation’s conditions during the CPB. First, the loss of pulsatile blood flow (both in non-pulsatile and pulsatile CPB pumps (Elbers et al., 2011; Elvevoll et al., 2016)) decreases the vascular resistance via loss of the myogenic autoregulation (Clifford, 2011) and lowers the pressure achieved within the circulatory system. Secondly, hemodilution associated with CPB decreases the Oxygen-Carrying Capacity of the Blood (CaO2), thus reducing the Delivery of Oxygen (DO2) to the tissues (DO2 is standardly indexed for body surface area–iDO2). Realizing this, it stands to reason that to prevent these adverse changes to the circulatory system, it is necessary to maintain adequate perfusion pressure and iDO2 at the same time. The obvious way to achieve this is to increase the flow of the CPB pump (Jufar et al., 2022), as it both raises the perfusion pressure and increases the iDO2 (iDO2 is a derivative of CaO2 and cardiac output, which in this case is replaced by the CPB pump flow).

To summarize, if MAP is maintained below the minimal kidney’s autoregulatory threshold during the CPB, it decreases the renal blood flow and drastically decreases medullary blood flow (efferent arteriole constriction with Ang II). Should iDO2 decrease as well (insufficient pump flow to compensate for a decreased CaO2), the kidneys will suffer additional ischemic damage, which can result in Cardiac Surgery Associated Acute Kidney Injury (CSA-AKI).

The main objective of this investigation was to assess the impact of increased MAP (achieved by increasing the CPB pump flow) on postoperative kidney function. Selected postoperative complications were also monitored to evaluate the impact of higher pressure on other organs.

## Materials and methods

One hundred nine adult patients were prospectively enrolled in this study. After receiving complete information regarding this investigation’s potential risks and benefits, each patient gave written consent to participate in the project. The investigation was conducted in accordance with the Declaration of Helsinki and Good Clinical Practice guidelines. The local Bioethical Committee’s approval was obtained prior to the patient’s recruitment (document’s signature: KB-0012/45/2021).

The intervention in this study was increasing the flow of the CPB pump to reach the target MAP of > 90 mmHg during the procedure. The control group had a standard pump flow of 2.4 L/min/m^2^. The pump flow in the study group was increased maximally to 150% of the standard flow rate. Catecholamines (adrenaline and noradrenaline) infusion was not a part of the intervention protocol in this study. Vasopressors were administered prior to CPB to maintain MAP > 70 mmHg. During the CPB, vasopressors were usually ceased, and their continuation depended on the decision of the doctor who provided the anesthesia (not an investigative team member). After the study enrolment, every patient was randomized into the study or control group. In order to prevent discrepancies between the groups, the randomization protocol included the patient’s gender and age. In the last year, the mean age of patients operated on in the facility where the study occurred was 69 years. The randomization scheme included two groups (<69 years of age and ≥ 69 years of age). Each group had two sub-groups of different gender. A series of letters, “A” or “B” (in equal amounts), was randomly generated for each of the four sub-groups using a computed signs generator. Consecutive patients enrolled in the study were randomly assigned to the study group (A) or the control group (B) within each sub-group based on age and gender.

As MAP does not maintain a constant value during the CPB, four MAP ranges were set for the whole study population. After the procedure, each patient was assigned to the MAP range that was dominant during his or her CPB. The ranges were as follows: < 55 mmHg, 56–70 mmHg, 71–90 mmHg, and ≥ 90 mmHg. As only one patient had the dominant CPB MAP of < 55 mmHg, this range was merged with the next one, leaving three MAP ranges in the ultimate result’s analysis: < 70 mmHg, 71–90 mmHg, and > 90 mmHg.

Blood and urine samples were taken from each patient at the following time points:

• Blood: before the operation, 6 h after weaning for the CPB
• Urine: before the operation, 6 h after weaning for the CPB, 24 h after the operation, 48 h after the operation, and 5 days after the operation

Blood was collected using S-Monovette 3.4 mL sterile containers (K3 EDTA: 1.6 mg/1 mL of blood; SARSTEDT AG & Co. KG Sarstedtstrasse 1, 51588 Nümbrecht, Germany). Urine was collected using standard non-sterile urine containers. After the collection, samples were stored at 5 C for no longer than 4 h and centrifuged (4°C, 10 min, 4000 RPM). After centrifugation, 1 mL of supernatant was taken and stored at −70°C.

The following kidney injury biomarkers’ concentration was measured in the samples:

• Blood: interleukin 6 (IL-6), interleukin 8 (IL-8), and tumor necrosis factor-alpha (TNF-α)
• Urine: neutrophil gelatinase-associated lipocalin (NGAL), kidney injury molecule 1 (KIM-1), matrix metalloproteinase 9 (MMP-9), and interleukin 18 (IL-18)

Quantitative assessment of IL-6, IL-8, and TNF-α (in plasma), as well as NGAL, KIM-1, IL-18, and MMP-9 (in urine) levels in patients enrolled in this study was performed using Luminex xMAP technology (Luminex Corporation, Austin, TX, United States). The concentrations of urine biomarkers were adjusted to creatinine excretion and presented in ng/mg of creatinine (NGAL/Cr, KIM-1/Cr, MMP-9/Cr, IL-18/Cr).

Plasma renin concentration was measured in the preoperative and 6 h after CPB blood samples, using standard ELISA kits (Demeditec Diagnostics GmbH, 24145 Kiel–Germany).

A preoperative creatinine clearance was measured using serum creatinine concentration and CKD-EPI formula. Early postoperative creatinine clearance (6 h after weaning from CPB) was calculated directly from serum and urine creatinine concentrations and urine volume. Urine output was monitored during the whole operation, and diuresis during the CPB was recorded separately.

Study inclusion criteria:

• Written consent to study enrolment
• Undergoing elective cardiac surgery procedure with the use of CPB
• Age at the time of enrolment ≥ 18 years

Study exclusion criteria:

• Postoperative complications involving increased inflammatory response (wound infection, pneumonia, sepsis) or possibly compromising cardiac output and CaO_2_ (myocardial infarct, severe blood loss, multi-organ failure)
• Kidney artery stenosis
• Active neoplasm
• Active inflammatory diseases
• KDIGO stage 5 kidney disease
• Postoperative hypotension (MAP < 70 mmHg)

All patients included in the study were monitored for AKI and other postoperative complications for 5 days after the operation. AKI was diagnosed according to the KDIGO criteria (Eknoyan et al., 2014): ≥ 0.3 mg/dL increase in serum creatinine, 50% increase in serum creatinine from the initial value, or diuresis < 0.5 mL/kg/h for at least 6 h. Long-term kidney function was assessed using serum creatinine measurement taken at least 3 months after the operation. The onset or progression of CKD was diagnosed according to KDIGO guidelines (Eknoyan et al., 2014).

All patients enrolled in this investigation were classified as ASA II–III. General anesthesia was induced with 100 µg of fentanyl, 0.3 mg/kg etomidate, and 0.6 mg/kg rocuronium. After endotracheal intubation, anesthesia was maintained with sevoflurane (MAC = 1.0). Analgesia was maintained with 250 µg fentanyl boluses every 30–40 min; an additional dose of 500 µg fentanyl was administered prior to sternotomy. Repeated doses of 0.2 mg/kg rocuronium were administered according to the patient’s requirements. After the initiation of normothermic CPB, mechanical ventilation was reduced to 50% of the initial tidal volume and 50% of the initial respiratory rate. Endotracheal sevoflurane administration was ceased, and volatile anesthesia was continued at the same rate via the membrane oxygenator of the CPB circuit.

Fisher exact test was used to compare qualitative variables between the groups. Since most of the quantitative variables showed distributions significantly different from a normal distribution (*p* < 0.05, Shapiro-Wilk test), non-parametric tests were used: Mann-Whitney test to compare values of rank and continuous variables between groups, and Spearman rank correlation coefficient to measure association between continuous variables. Statistica 13 was used to perform the calculations. *p* < 0.05 was considered statistically significant.

## Results

**SCHEME 1:**
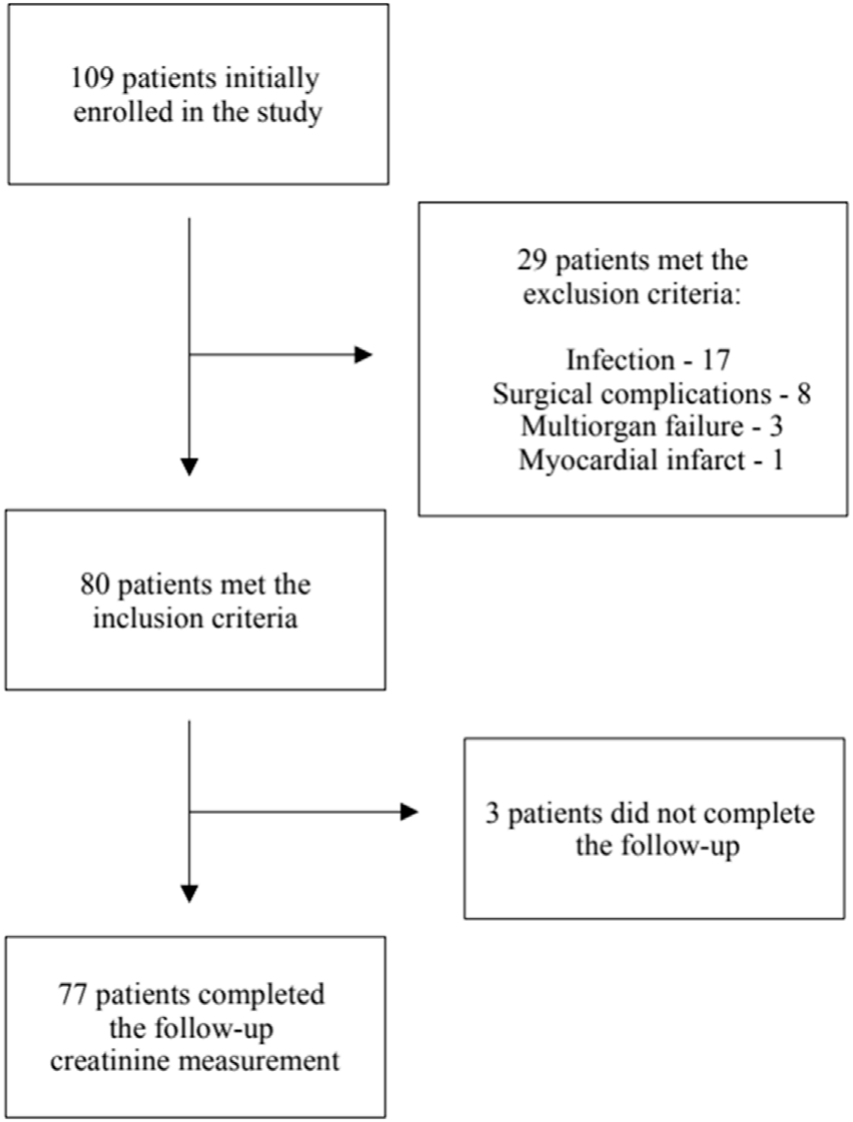
Patient enrolment in the study.

Twenty nine patients met the exclusion criteria, leaving eighty patients for the final analysis. The follow-up creatinine measurement was unavailable in three patients–Scheme 1.

There were no significant differences in age, gender, comorbidities, or initial laboratory results between the patients from the included and the excluded groups. The only differences included the Euro Score Logistic value and percentage of complex procedures, which were both higher in the excluded patient group–Supplementary Table S1.

The study (A) and control (B) groups did not differ in terms of age, gender, comorbidities, or initial laboratory results. Patients in the study group maintained higher hematocrit values during the CPB. With greater CPB pump flow, there was a higher iDO_2_ in this patient group. There were no differences in postoperative complications between the study and control group. Target MAP of > 90 mmHg was achieved in 31.82% of the study group and 8.33% of the control group. In the control group, the dominant MAP range was 70–90 mmHg (50%), the same as in the study group (52.27%). There was a relatively high percentage of MAP < 70 mmHg in the control group (41.67%) compared to the study group (15.91%). Results are summarized in Table 1.

**TABLE 1:** Comparison of the study (A) and control (B) group.

		Study group (A) (*n* = 44)	Control group (B) (*n* = 36)	*p*-value*
Age [years] mean ± SD (M)		66.52 ± 8.17 (68)	67.92 ± 8.02 (69.5)	0.375
Gender n, (%)	Female	10 (23%)	10 (28%)	0.615
	Male	34 (77%)	26 (72%)	
BMI mean ± SD (M)		28.63 ± 3.69 (28.2)	27.36 ± 3.78 (27.3)	0.283
ESL mean ± SD (M)		3.59 ± 2.56 (2.9)	3.99 ± 3.11 (3.5)	0.498
Ht_0_ [%] mean ± SD (M)		41.06 ± 3.18 (41.7)	40.49 ± 3.56 (40.8)	0.411
eGFR_0_ [ml/min/1.73m^2^] mean ± SD (M)		79.57 ± 15.89 (80.5)	73.83 ± 20.98 (74)	0.223
Hypertension n, (%)		38 (86%)	26 (72%)	0.161
Diabetes n, (%)		19 (43%)	11 (31%)	0.353
CKD n, (%)		3 (7%)	6 (17%)	0.286
Dyslipidemia n, (%)		25 (57%)	20 (56%)	1
Operation n, (%)	CABG	25 (57%)	24 (67%)	0.489
	Valvular	5 (11%)	7 (19%)	0.359
	CABG + valvular	11 (25%)	3 (8%)	0.075
	Complex procedures	3 (7%)	2 (6%)	1
Total CPB time mean ± SD (M) [min]		77.84 ± 33.74 (72.5)	71.67 ± 34.75 (59.5)	0.312
Aortic cross-clamp time mean ± SD (M) [min]		55.02 ± 28.03 (48.5)	47.19 ± 26.68 (37.5)	0.169
Mean CPB pump flow mean ± SD (M) [l/min]		5.38 ± 0.59 (5.3)	4.92 ± 0.56 (5)	**0.002**
Mean Ht_CPB_1 mean ± SD (M) [%]		26.93 ± 3.81 (27)	25.03 ± 4.63 (24)	**0.028**
Mean Ht_CPB_2 mean ± SD (M) [%]		29.80 ± 3.65 (29.5)	27.74 ± 3.23 (27)	**0.007**
Mean iDO_2_ during the CPB mean ± SD (M) [ml/min/m^2^]		334.94 ± 46.23 (328.8)	301.40 ± 46.86 (297.6)	**0.001**
CSA-AKI n, (%)		12 (27%)	14 (39%)	0.339
Postoperative cerebral stroke n, (%)		0 (0%)	1 (3%)	0.450
Postoperative TIA n, (%)		1 (2%)	0 (0%)	1
Postoperative delirium n, (%)		5 (11%)	9 (25%)	0.143
Onset or progression of CKD n, (%)		1 (2%)	3 (9%)	0.316
eGFR after 3 months mean ± SD (M) [ml/min/1.73m^2^]		82.98 ± 16.15 (82)	82.32 ± 19.76 (83.5)	0.882

BMI, body mass index; CABG, coronary artery bypassing graft; CKD, chronic kidney disease; CPB, cardiopulmonary bypass; CSA-AKI, cardiac surgery-associated acute kidney injury, eGFR_0_, preoperative estimated glomerular filtration rate; ESL, EuroSCORE logistic; Ht_0_, preoperative hematocrit value; Ht_CPB_1, first hematocrit value during the CPB; Ht_CPB_2, second hematocrit value during the CPB; iDO2, oxygen delivery indexed for body surface area; M, median value; SD, standard deviation; TIA, transient ischemic attack; *, calculated using Mann-Whitney test for quantitative variables and Fisher exact test for qualitative variables. *p* values < 0.05 are put in bold.

As many patients in the study group did not reach the target MAP of > 90 mmHg, the primary outcomes analysis focused on patients from different MAP ranges and those who eventually developed and did not develop CSA-AKI.

The patients who suffered from CSA-AKI had worse preoperative kidney function, which was also demonstrated with higher preoperative biomarkers’ concentration. The percentage of complex procedures and the postoperative use of Noradrenaline (NA) were higher in this group. CSA-AKI was associated with the onset or progression of CKD in this study population, and also with higher postoperative biomarkers concentration. No other significant differences were observed–Table 2.

**TABLE 2:** Comparison of AKI vs. no-AKI groups.

		AKI (*n* = 26)	no-AKI (*n* = 54)	*p*-value*
Age [years] mean ± SD (M)		66.54 ± 8.10 (71)	66.48 ± 8.06 68)	0.239
Gender n, (%)	Female	8 (31%)	12 (22%)	0.421
	Male	18 (69%)	42 (78%)	
BMI mean ± SD (M)		27.92 ± 3.94 (27.3)	28.12 ± 3.71 (27.9)	0.873
ESL mean ± SD (M)		4.11 ± 2.52 (3.9)	3.61 ± 2.95 (2.8)	0.208
Ht_0_ [%] mean ± SD (M)		40.31 ± 3.84 (41.5)	41.04 ± 3.09 (41.5)	0.813
Hb_A1C_ [%] mean ± SD (M)		6.30 ± 0.89 (6)	6.18 ± 0.70 (6)	0.789
eGFR_0_ [ml/min/1.73 m^2^] mean ± SD (M)		69.58 ± 20.88 (68)	80.56 ± 16.20 (81)	**0.025**
Hypertension n, (%)		23 (88%)	41 (76%)	0.242
Diabetes n, (%)		11 (42%)	19 (35%)	0.624
CKD n, (%)		6 (23%)	3 (6%)	0.052
Dyslipidemia n, (%)		13 (50%)	32 (59%)	0.477
Preoperative NGAL/Cr mean ± SD (M) [ng/mg]		11.82 ± 24.74 (4.5)	5.66 ± 11.20 (2.9)	**0.024**
Preoperative IL-18/Cr mean ± SD (M) [ng/mg]		0.071 ± 0.047 (0.06)	0.049 ± 0.043 (0.04)	**0.013**
Preoperative MMP-9/Cr mean ± SD (M) [ng/mg]		8.91 ± 39.74 (0.09)	2.48 ± 13.55 (0.02)	**0.006**
Operation n, (%)	CABG	12 (46%)	37 (69%)	0.085
	Valvular	4 (15%)	8 (15%)	1
	CABG + valvular	6 (23%)	8 (15%)	0.366
	Complex procedures	4 (15%)	1 (2%)	**0.036**
Total CPB time mean ± SD (M) [min]		81.85 ± 34.96 (86.5)	71.80 ± 33.55 (60.5)	0.214
Aortic cross-clamp time mean ± SD (M) [min]		57.77 ± 29.64 (63)	48.48 ± 26.21 (40.5)	0.252
Postoperative noradrenaline dose mean ± SD (M) [mg/kg]		0.057 ± 0.078 (0.031)	0.042 ± 0.164 (0)	**0.005**
IL-8 6 h after the CPB mean ± SD (M)[pg/ml]		22.26 ± 19 (17)	14.79 ± 10.16 (11.9)	**0.045**
TNF-ɑ 6 h after the CPB mean ± SD (M) [pg/ml]		9.18 ± 4.14 (8.2)	6.40 ± 2.19 (5.9)	**<0.001**
NGAL/Cr 6 h after the CPB mean ± SD (M) [ng/mg]		16.98 ± 29.44 (5)	8.10 ± 28.22 (3.4)	**0.014**
NGAL/Cr 24 h after the operation mean ± SD (M) [ng/mg]		32.21 ± 37.20 (26)	18.94 ± 12.98 (16.6)	**0.025**
IL-18/Cr 24 h after the operation mean ± SD (M) [ng/mg]		0.492 ± 0.457 (0.3)	0.378 ± 0.406 (0.2)	**0.029**
Onset or progression of CKD n, (%)		4 (17%)	0 (0%)	**0.008**
eGFR after 3 months mean ± SD (M) [ml/min/1.7 m^2^]		70.04 ± 22.01 (71.5)	86.60 ± 13.94 (85)	**0.020**

BMI, body mass index; CABG, coronary artery bypassing graft; CKD, chronic kidney disease; CPB, cardiopulmonary bypass; eGFR_0_, preoperative estimated glomerular filtration rate; ESL, EuroSCORE logistic; Hb_A1C_, preoperative glycated hemoglobin percentage; Ht_0_, preoperative hematocrit value; IL-8, serum interleukin 8 concentration; IL-18/Cr, serum interleukin 18 concentration; M, median value; MMP-9, urine matrix metalloproteinase 9 concentration normalized for creatinine excretion; NGAL/Cr, urine neutrophil gelatinase-associated lipocalin concentration normalized for creatinine excretion; SD, standard deviation; TNF-ɑ, serum tumor necrosis factor-alpha concentration; *, calculated using Mann-Whitney test for quantitative variables and Fisher exact test for qualitative variables. *p* values < 0.05 are put in bold.

MAP decreased in 78.75% of the study population immediately after the initiation of CPB. The average decrease in MAP was 14.38%. Patients with higher MAP after the induction of general anesthesia had higher MAP during the CPB. The highest MAP maintained during CPB in this investigation was 110 mmHg.

Maintaining a higher MAP during CPB in this study population did not affect CSA-AKI incidence. It did, however, increase the intraoperative and postoperative diuresis, as well as decreased renin release associated with CPB. On the other hand, patients in the lowest MAP group had greater CaO2 and iDO2 decrease as well as higher NA demand compared to patients in the highest MAP group. Patients in the higher MAP groups were older, had higher BMI, higher preoperative urine KIM-1/Cr concentration, and statistically more women were amongst them. Patients with lower MAP during CPB had a greater decrease in creatinine clearance 6 h after the surgery, but the result was not statistically significant. Higher MAP during the CPB did not increase the incidence of cerebrovascular complications after the operation; patients in the highest MAP group had the smallest percentage of complications, but the difference was not statistically significant–Table 3.

**TABLE 3:** Comparison of patients subjected to different MAP ranges during the CPB. Each MAP range was coded with a letter (C–E) for more transparent data presentation.

			C	D	E	*p*-value*		
			MAP: < 70 mmHg	MAP: 70–90 mmHg	MAP: > 90 mmHg	C vs. D	C vs. E	D vs. E
Age [years] mean ± SD (M)			64.45 ± 8.14 (67)	68.22 ± 7.99 (69)	68.06 ± 7.88 (69)	**0.049**	0.178	0.837
Gender n, (%)		Female	2 (9%)	11 (27%)	7 (41%)	**0.020**		
		Male	20 (91%)	30 (73%)	10 (59%)			
BMI mean ± SD (M)			26.96 ± 3.98 (27)	27.98 ± 3.78 (27)	29.67 ± 2.98 (29)	0.569	**0.039**	**0.043**
ESL mean ± SD (M)			3.33 ± 2.48 (2.8)	3.67 ± 2.92 (2.8)	4.57 ± 2.93 (3.9)	0.584	0.113	0.245
Ht_0_ [%] mean ± SD (M)			40.53 ± 2.91 (41.2)	40.99 ± 3.33 (41.2)	40.71 ± 4.03 (41.8)	0.639	0.552	0.993
eGFR_0_ mean ± SD (M) [ml/min/1.73m^2^]			76.27 ± 20.64 (75.5)	79.49 ± 15.89 (80)	74.29 ± 21.85 (79)	0.512	0.966	0.489
CKD n, (%)			2 (9%)	4 (10%)	3 (18%)	0.442		
Preoperative KIM-1/Cr mean ± SD (M) [ng/mg]			0.756 ± 1.147 (0.25)	0.562 ± 0.701 (0.32)	1.620 ± 2.657 (0.80)	0.751	0.079	**0.021**
Operation n, (%)	CABG		12 (55%)	25 (61%)	12 (71%)	0.321		
	Valvular		3 (14%)	8 (20%)	1 (6%)	0.604		
	CABG + valvular		4 (18%)	6 (15%)	4 (24%)	0.731		
	Complex procedures		3 (14%)	2 (5%)	0 (0%)	0.074		
Total CPB time mean ± SD (M) [min]			83.86 ± 37.11 (73.5)	71.02 ± 29.78 (71)	73.41 ± 39.66 (58)	0.136	0.315	0.925
Aortic cross-clamp time mean ± SD (M) [min]			57.32 ± 31.11 (48)	49.39 ± 25.17 (43)	49.06 ± 28.73 (42)	0.296	0.590	0.912
MAP after the induction of general anesthesia mean ± SD (M) [mmHg]			84.68 ± 12.30 (83)	90.61 ± 12.14 (93)	93.88 ± 8.14 (96)	**0.040**	**0.007**	0.434
Intraoperative noradrenaline dosemean ± SD (M) [mg/kg]			0.012 ± 0.010 (0.01)	0.006 ± 0.006 (0.01)	0.005 ± 0.006 (0)	**0.008**	**0.008**	0.532
Diuresis during the CPB mean ± SD (M) [ml/kg/h]			2.90 ± 1.78 (2.38)	3.87 ± 2.72 (3.48)	4.97 ± 2.89 (4.54)	0.198	**0.006**	0.107
Δ CaO_2_ mean ± SD (M) [%]			63.19 ± 7.27 (63.9)	65.82 ± 8.41 (66.3)	68.15 ± 8.08 (66.3)	**0.040**	**0.036**	0.688
Mean iDO_2_ during the CPB mean ± SD (M) [ml/min/m^2^]			305.90 ± 44.44 (305)	319.47 ± 52.90 (315)	338.79 ± 41.27 (335)	0.299	**0.002**	0.114
Δ Renin M (Q1-Q3) [%]			364.28 (213.99–4.95.26	286.28 (163.82–532.99)	166.89 (101.59–266.33)	0.428	**0.008**	**0.048**
Δ Creatinine_C_ mean ± SD (M) [%]			77.68 ± 48.61 (74.6)	74.70 ± 46.48 (67.4)	98.57 ± 67.10 (91.5)	0.719	0.350	0.135
CSA-AKI n, (%)			6 (27%)	16 (39%)	4 (24%)	0.929		
Postoperative cerebral stroke n, (%)			1 (5%)	0 (0%)	0 (0%)	0.171		
Postoperative TIA n, (%)			0 (0%)	1 (2%)	0 (0%)	0.907		
Postoperative delirium n, (%)			7 (32%)	5 (12%)	2 (12%)	0.077		

BMI, body mass index; CABG, coronary artery bypassing graft; CKD, chronic kidney disease; CPB, cardiopulmonary bypass; CSA-AKI, cardiac surgery associated acute kidney injury, eGFR0, preoperative estimated glomerular filtration rate; ESL, EuroSCORE logistic, Ht0, preoperative hematocrit value; iDO2, oxygen delivery indexed for body surface area; KIM-1/Cr, urine kidney injury molecule 1 concentration normalized for creatinine excretion; M, median value; MAP, mean arterial pressure; SD, standard deviation; TIA, transient ischemic attack, Q1-Q3, first and third quartile values; Δ CaO2, delta of preoperative and mean intraoperative blood oxygen-carrying capacity; Δ CreatinineC, delta of 6-hour-postoperative and preoperative creatinine clearance; Δ Renin, delta of 6-hour-postoperative and preoperative plasma renin concentration; *, calculated using Mann-Whitney test for differences in quantitative variables between the MAP groups, and also for associations of MAP range treated as rank variable with qualitative variables. *p* values < 0.05 are put in bold.

There was a negative correlation between diuresis during the CPB and perioperative serum creatinine concentrations, as well as the follow-up creatinine concentration–Table 4.

**TABLE 4:** Correlation between diuresis during the CPB and perioperative and follow-up serum creatinine concentration.

	Preoperative serum creatinine	Creatinine_1_	Creatinine_2_	Creatinine_3_	Creatinine_4_	Follow-up serum creatinine
Diuresis during the CPB	R = −0.256 *p* = 0.026	R = −0.327 *p* = 0.004	R = −0.319 *p* = 0.005	R = −0.317 *p* = 0.005	R = −0.271 *p* = 0.039	R = −0.243 *p* = 0.038

CPB, cardiopulmonary bypass; Creatinine_1-4_, consecutive postoperative serum creatinine measurements; p, *p*-value (calculated using Mann-Whitney test); R, Spearman’s correlation coefficient.

## Discussion

This investigation aimed to determine whether a higher perfusion pressure during CPB can alleviate kidney injury associated with this procedure. The obtained results suggest that it can. Mayor benefits of maintaining high MAP during the CPB included greater diuresis and decreased renin increase after the operation. Diuresis is a direct indicator of kidney function (Yao and Gao, 2021), and a lower renin increase proves reduced kidney hypoperfusion (Küllmar et al., 2021). Greater urine output in the higher MAP groups could be partially attributed to a smaller decrease in CaO2 and iDO2 during the CPB. However, renin release is irrespective of these two parameters and depends solely on perfusion pressure. When the RAA axis response is triggered within the juxtaglomerular cells of the afferent arteriole, it means that the current MAP is below the kidneys’ autoregulatory range. The kidneys strive to raise the systemic pressure and maintain glomerular filtration pressure at the expense of peritubular and medullary blood flow. Thus, there is a tight dependency between renin release and reduced blood flow to the post-glomerular capillaries, which can lead to ischemic kidney injury. Lower renin increase after the operation indicates that kidneys received more adequate perfusion and endured less ischemic stress.

There was no statistically significant difference in CSA-AKI incidence between the different MAP groups, which can be attributed to the modest sample size in this study. Comparing these results with the results of other authors is difficult, as higher MAP values in these studies were achieved using NA infusion (Azau et al., 2014; Vedel et al., 2018; De La et al., 2022), which can have an adverse impact on the kidneys (Huette et al., 2022). There is, however, scientific evidence that increasing CPB pump flow can improve renal blood flow during the CPB (Lee et al., 2020). Such an approach is all the more convincing as it mimics the physiological circulatory response to decreased CaO2, which is increasing the cardiac output. The authors of this study did not encounter any scientific investigations where target MAP during the CPB was achieved by increasing the pump flow. There are, however, reports that higher iDO2 during the CPB (achieved by increasing the pump flow) can reduce the risk of CSA-AKI (Mukaida et al., 2019; de et al., 2011). Taking advantage of the dual benefit of increasing the CPB pump flow (simultaneous MAP and iDO2 adjustment) appears optimal for preserving kidney function.

Determining an optimal MAP range for kidney function during the CPB is a complicated issue. As was mentioned in the Introduction section, under physiological conditions, the lower autoregulatory threshold of renal blood flow is 70–80 mmHg. In this investigation, the most distinct differences in outcomes can be noticed between the lowest (<70 mmHg) and the highest (>90 mmHg) MAP groups. This suggests that MAP < 70 mmHg is disadvantageous to the kidneys, while MAP > 90 mmHg is sufficient to maintain kidney function. The results in the middle MAP group (70–90 mmHg) vary, which suggests that the lower kidney autoregulatory threshold during CPB is somewhere in this MAP range.

Novel kidney injury biomarkers (IL-6, IL-8, TNF-α, NGAL, KIM-1, MMP-9, and IL-18) were used in this investigation to assess kidney damage more accurately. They proved efficient in the early detection of CSA-AKI, as there were significant differences in their postoperative concentration between the AKI and no-AKI groups. There was no significant difference in postoperative biomarkers’ concentration between the different MAP groups. Several reasons can explain this result, such as the limited study population or the unknown hydration status of the enrolled patients.

There was a constant negative correlation between diuresis during the CPB and postoperative serum creatinine concentration. This demonstrates that better intraoperative kidney function (indicated by greater diuresis) directly impacts early postoperative kidney function. Hence, maintaining higher MAP during the CPB improves the kidney’s filtration function after the surgery.

Some patients in this investigation who suffered from CSA-AKI experienced the onset or progression of CKD. The impact of AKI on patients’ morbidity and mortality is well established (Harty; Gameiro et al., 2020), and so nephroprotection during cardiac surgeries is of great importance. It is worth mentioning that diuresis during the CPB (enhanced by MAP > 90 mmHg) presented a significant negative correlation with serum creatinine concentration after 3 months. This suggests a positive long-term impact of higher perfusion pressure on postoperative kidney function.

The analysis of cerebrovascular complications proved that MAP values > 90 mmHg during the CPB are safe for the central nervous system. The same applies to the increased CPB pump flow, as there was no higher complication rate in the study group, which had a significantly greater pump flow. Looking at the incidence of postoperative delirium (Table 3), it is justified to state that increasing MAP during the CPB could decrease the risk of this complication. These findings are consistent with other researchers’ discoveries (Siepe et al., 2011; Brown et al., 2019).

This study had several limitations. Its primary limitation is a modest sample size. The statistical power of our study with 44 and 36 subjects in the study and control group respectively was sufficient to detect the real differences between the groups with 80% probability. This corresponds to 0.64 standard deviations of the studied parameters. Smaller differences could remain undetected. Furthermore, this study population had no set fluid intake regimen. The patients were allowed to intake fluids at will on the day preceding the operation. Only a dominant MAP range during the CPB was recorded during this investigation. Due to technical reasons, there was no continuous recording of MAP or pump flow, which could provide valuable data for this study.

## Conclusions

Maintaining MAP > 90 mmHg during the CPB positively impacts intraoperative and postoperative kidney function. It significantly reduces renal hypoperfusion during the procedure compared to MAP < 70 mmHg. MAP > 90 mmHg is safe for the central nervous system, and preliminary results suggest that it may have a beneficial impact on the incidence of postoperative delirium.

## Data Availability

The raw data supporting the conclusions of this article will be made available by the authors, without undue reservation.

## References

[B1] Azau A. Markowicz P. Corbeau J. J. Cottineau C. Moreau X. Baufreton C. (2014). Increasing mean arterial pressure during cardiac surgery does not reduce the rate of postoperative acute kidney injury. Perfus. (United Kingdom) 29 (6), 496–504. 10.1177/0267659114527331 24619062

[B2] Brown C. H. Neufeld K. J. Tian J. Probert J. Laflam A. Max L., (2019). Effect of targeting mean arterial pressure during cardiopulmonary bypass by monitoring cerebral autoregulation on postsurgical delirium among older patients: a nested randomized clinical trial. JAMA Surg 154 (9), 819–826. 10.1001/jamasurg.2019.1163 31116358 PMC6537779

[B3] Burke M. Pabbidi M. Farley J. Roman R. (2014). Molecular mechanisms of renal blood flow autoregulation. Current Vascular Pharmacology 12 (6), 845–858. 10.2174/15701611113116660149 24066938 PMC4416696

[B4] Clifford P. S. (2011). Local control of blood flow. Advances in Physiology Education 35 (1), 5–15. 10.1152/advan.00074.2010 21385995

[B5] De La Hoz M. A. Rangasamy V. Bastos A. B. Xu X. Novack V. Saugel B., (2022). Intraoperative hypotension and acute kidney injury, stroke, and mortality during and outside cardiopulmonary bypass: a retrospective observational cohort study. Anesthesiology 136 (6), 927–939. 10.1097/ALN.0000000000004175 35188970

[B6] de Somer F. Mulholland J. W. Bryan M. R. Aloisio T. Van Nooten G. J. Ranucci M. (2011). O2delivery and CO2production during cardiopulmonary bypass as determinants of acute kidney injury: time for a goal-directed perfusion management? Critical Care 15 (4), R192. 10.1186/cc10349 21831302 PMC3387634

[B7] Eknoyan G. Norbert Lameire M. D. PhD F. K. C.-C. (2014). KDIGO 2012 clinical practice guideline for the evaluation and management of chronic kidney disease. IFAC Proc. Vol. (IFAC-PapersOnline) 19 (1), 4477–4483. 10.3182/20140824-6-za-1003.01333

[B8] Elbers P. W. G. Wijbenga J. Solinger F. Yilmaz A. Van Iterson M. Van Dongen E. P. A., (2011). Direct observation of the human microcirculation during cardiopulmonary bypass: effects of pulsatile perfusion. Journal of Cardiothoracic and Vascular Anesthesia 25 (2), 250–255. 10.1053/j.jvca.2010.06.014 20800509

[B9] Elvevoll B. Lundemoen S. Svendsen Ø. S. Mongstad A. Grong K. Kvalheim V. L., 2016. Does roller pump–induced pulsatile CPB perfusion affect microvascular fluid shifts and tissue perfusion? The Annals of Thoracic Surgery 102 (2), 564–572. 10.1016/j.athoracsur.2016.02.005 27139370

[B10] Gameiro J. Fonseca J. A. Outerelo C. Lopes J. A. (2020). Acute kidney injury: from diagnosis to prevention and treatment strategies. Journal of Clinical Medicine 9 (6), 1704. 10.3390/jcm9061704 32498340 PMC7357116

[B11] Harty J. Prevention and management of acute kidney injury. uk. https://www.ums.ac. PMC4255835 25484464

[B12] Huette P. Moussa M. D. Beyls C. Guinot P. G. Guilbart M. Besserve P., (2022). Association between acute kidney injury and norepinephrine use following cardiac surgery: a retrospective propensity score-weighted analysis. Annals of Intensive Care 12 (1), 61–69. 10.1186/s13613-022-01037-1 35781575 PMC9250911

[B13] Jefferson J. A. Thurman J. M. Schrier R. W. (2010). Pathophysiology and etiology of acute kidney injury. Fourth Edi. Elsevier Inc. Amsterdam, The Netherlands, 10.1016/B978-0-323-05876-6.00066-6

[B14] Jufar A. H. Lankadeva Y. R. May C. N. Cochrane A. D. Marino B. Rinaldo Bellomo R. G. E. Renal and cerebral hypoxia and inflammation during cardiopulmonary bypass. Comprehensive Physiology 12, 2022, 2799 (1). 10.1002/cphy.c210019 34964119

[B15] Kennedy-Lydon T. M. Crawford C. Wildman S. S. P. Peppiatt-Wildman C. M. (2013). Renal pericytes: regulators of medullary blood flow. Acta Physiologica 207 (2), 212–225. 10.1111/apha.12026 23126245 PMC3561688

[B16] Kotani Y. Kataoka Y. Izawa J. Fujioka S. Yoshida T. Kumasawa J., (2022). High versus low blood pressure targets for cardiac surgery while on cardiopulmonary bypass. Cochrane Database Systematic Reviews 2022 (11). 10.1002/14651858.CD013494.pub2 PMC9709767 36448514

[B17] Küllmar M. Saadat-Gilani K. Weiss R. Massoth C. Lagan A. Cortés M. N., (2021). Kinetic changes of plasma renin concentrations predict acute kidney injury in cardiac surgery patients, Obs. Study, 203. 10.1164/rccm.202005-2050OC 33320784

[B18] Kunst G. Milojevic M. Boer C. De Somer F. M. J. J. Gudbjartsson T. van den Goor J., (2019). 2019 EACTS/EACTA/EBCP guidelines on cardiopulmonary bypass in adult cardiac surgery. British Journal of Anaesthesia 123 (6), 713–757. 10.1016/j.bja.2019.09.012 31585674

[B19] Lappin J. H. F. S. L. Physiology, renin Angiotensin system. StatPearls. https://www.ncbi.nlm.nih.gov/books/NBK470410/#_ncbi_dlg_citbx_NBK470410. (Accessed May 20, 2023). 29261862

[B20] Lee C. J. Gardiner B. S. Smith D. W. (2020). A cardiovascular model for renal perfusion during cardiopulmonary bypass surgery. Comput. Biol. Med. 119 , 103676. 10.1016/j.compbiomed.2020.103676 32339121

[B21] Mukaida H. Matsushita S. Kuwaki K. Inotani T. Minami Y. Saigusa A., (2019). Time–dose response of oxygen delivery during cardiopulmonary bypass predicts acute kidney injury. J. Thorac. Cardiovasc. Surg. 158 (2), 492–499. 10.1016/j.jtcvs.2018.10.148 30578056

[B22] Navar L. G. Arendshorst W. J. Pallone T. L. Inscho E. W. Imig J. D. Bell P. D. (2008). The renal microcirculation. Microcirculation, Academic Press, Cambridge, MA, USA, 550–683. 10.1016/B978-0-12-374530-9.00015-2

[B23] Siepe M. Pfeiffer T. Gieringer A. Zemann S. Benk C. Schlensak C., (2011). Increased systemic perfusion pressure during cardiopulmonary bypass is associated with less early postoperative cognitive dysfunction and delirium. Eur. J. Cardio-thoracic Surg. 40 (1), 200–207. 10.1016/j.ejcts.2010.11.024 21168339

[B24] Vedel A. G. Holmgaard F. Rasmussen L. S. Langkilde A. Paulson O. B. Lange T., (2018). High-target versus low-target blood pressure management during cardiopulmonary bypass to prevent cerebral injury in cardiac surgery patients: a randomized controlled trial. Circulation 137 (17), 1770–1780. 10.1161/CIRCULATIONAHA.117.030308 29339351

[B25] Yao Y. L. Gao Y. (2021). Present situation and research progress of kidney function recoverability evaluation of acute kidney injury patient. International Journal of General Medicine. 14, 1919–1925. 10.2147/IJGM.S303348 34040424 PMC8140891

