# Supplementary material for: Higher perfusion pressure and pump flow during cardiopulmonary bypass are beneficial for kidney function–a single-centre prospective study
Source: Front Physiol. 2024 Feb 14;15:1257631. doi: 10.3389/fphys.2024.1257631 (PMC10899324; doi:10.3389/fphys.2024.1257631)
Supplement: Supplementary file 1 [file Table1.pdf]

<u>Supplementary Table 1</u>. Comparison of patients included in and excluded from the study.

		**Included**(n=80)	**Excluded** (n=29)	p-value*
Age [years]mean ± SD		67.15 ± 8.08	66.76 ± 9.53	0.871
Gendern, (%)	Female	20 (25%)	5 (17%)	0.451
	Male	60 (75%)	24 (83%)	
BMImean ± SD		28.06 ± 3.76	27.83 ± 3.20	0.789
ESLmean ± SD		3.77 ± 2.81	5.44 ± 3.73	**0.017**
Ht_0 [%]mean ± SD		40.81 ± 3.35	40.86 ± 3.53	0.964
Hb_{A1C} [%]mean ± SD		6.21 ± 0.76	6.05 ± 0.68	0.530
$eGFR_0$ [ml/min/1.73m^2]mean ± SD		76.99 ± 18.46	77.14 ± 14.40	0.829
$CK\text{-}MB_0$ [U/l]mean ± SD		17.83 ± 6.30	17.90 ± 6.95	0.961
Hypertensionn, (%)		64 (80%)	20 (69%)	0.302
Diabetesn, (%)		36 (38%)	6 (21%)	0.112
CKDn, (%)		9 (11%)	3 (10%)	1
Dyslipidemian, (%)		45 (56%)	18 (62%)	0.664
Stroken, (%)		5 (6%)	3 (10%)	0.436
TIAn, (%)		1 (1%)	0 (0%)	1
Carotid artery stenosisn, (%)		11 (14%)	4 (14%)	1
Operationn, (%)	CABG	49 (61%)	14 (48%)	0.275
	Valvular	12 (15%)	3 (10%)	0.755

	CABG + valvular	14 (18%)	4 (14%)	0.776
	Complex procedures	5 (6%)	8 (28%)	**0.005**

Legend: BMI – body mass index, CABG – coronary artery bypassing graft, CKD – chronic kidney disease, $CK\text{-}MB_0$ – preoperative creatine kinase MB isoenzyme level, $eGFR_0$– preoperative estimated glomerular filtration rate, ESL – EuroSCORE Logistic, Hb_{A1C} – preoperative glycated hemoglobin percentage, Ht_0 – preoperative hematocrit value, TIA – transient ischemic attack, SD – standard deviation, * - calculated using Mann-Whitney test for quantitative variables and Fisher exact test for qualitative variables.
